# Free-breathing high resolution modified Dixon steady-state angiography with compressed sensing for the assessment of the thoracic vasculature in pediatric patients with congenital heart disease

**DOI:** 10.1186/s12968-021-00810-8

**Published:** 2021-10-25

**Authors:** Narine Mesropyan, Alexander Isaak, Darius Dabir, Christopher Hart, Anton Faron, Christoph Endler, Dmitrij Kravchenko, Christoph Katemann, Claus C. Pieper, Daniel Kuetting, Ulrike I. Attenberger, Julian A. Luetkens

**Affiliations:** 1 Department of Diagnostic and Interventional Radiology, University Hospital Bonn, Venusberg- Campus 1, 53127 Bonn, Germany; 2 Quantitative Imaging Lab Bonn (QILaB), Venusberg-Campus 1, 53127 Bonn, Germany; 3 Department of Pediatric Cardiology, University Hospital Bonn, Venusberg-Campus 1, 53127 Bonn, Germany; 4 Philips Healthcare, Hamburg, Germany

**Keywords:** Congenital heart disease, Magnetic resonance angiography, Steady-state, Modified Dixon, Cardiovascular magnetic resonance

## Abstract

**Background:**

Cardiovascular magnetic resonance angiography (CMRA) is a non-invasive imaging modality of choice in pediatric patients with congenital heart disease (CHD). This study was aimed to evaluate the diagnostic utility of a respiratory- and electrocardiogram-gated steady-state CMRA with modified Dixon (mDixon) fat suppression technique and compressed sensing in comparison to standard first-pass CMRA in pediatric patients with CHD at 3 T.

**Methods:**

In this retrospective single center study, pediatric CHD patients who underwent CMR with first-pass CMRA followed by mDixon steady-state CMRA at 3 T were analyzed. Image quality using a Likert scale from 5 (excellent) to 1 (non-diagnostic) and quality of fat suppression were assessed in consensus by two readers. Blood-to-tissue contrast and quantitative measurements of the thoracic vasculature were assessed separately by two readers. CMRA images were reevaluated by two readers for additional findings, which could be identified only on either one of the CMRA types. Paired Student *t* test, Wilcoxon test, and intraclass correlation coefficients (ICCs) were used for statistical analysis.

**Results:**

32 patients with CHD (3.3 ± 1.7 years, 13 female) were included. Overall image quality of steady-state mDixon CMRA was higher compared to first-pass CMRA (4.5 ± 0.5 vs. 3.3 ± 0.5; P < 0.001). Blood-to-tissue contrast ratio of steady-state mDixon CMRA was comparable to first-pass CMRA (7.85 ± 4.75 vs. 6.35 ± 2.23; P = 0.133). Fat suppression of steady-state mDixon CMRA was perfect in 30/32 (94%) cases. Vessel diameters were greater in first-pass CMRA compared to steady-state mDixon CMRA with the greatest differences at the level of pulmonary arteries and veins (e.g., right pulmonary artery for reader 1: 10.4 ± 2.4 vs. 9.9 ± 2.3 mm, P < 0.001). Interobserver agreement was higher for steady-state mDixon CMRA for all measurements compared to first-pass CMRA (ICCs > 0.92). In 9/32 (28%) patients, 10 additional findings were identified on mDixon steady-state CMRA (e.g., partial anomalous venous return, abnormalities of coronary arteries, subclavian artery stenosis), which were not depicted using first-pass CMRA.

**Conclusions:**

Steady-state mDixon CMRA offers a robust fat suppression, a high image quality, and diagnostic utility for the assessment of the thoracic vasculature in pediatric CHD patients.

## Background

Over the last decades, surgical, interventional and supportive care in patients with congenital heart disease (CHD) have undergone significant improvements from conservative to highly specialized therapeutic strategies [1, 2]. This led not only to longer life expectancy, but also allowed to achieve a better quality of life in this patient population [3, 4]. However, this achievement is based not only on the therapeutic advances of recent years. In particular, advances in non-invasive imaging allow earlier and more accurate diagnosis and monitoring of CHD patients today [5–7].

In this regard, cardiovascular magnetic resonance (CMR) including cardiovascular magnetic resonance angiography (CMRA) has also experienced a fast evolution and now represents not only the imaging standard, but also an important pillar in terms of risk stratification and procedural planning [7–9]. Furthermore, CMR including CMRA has proven to be a reliable and accurate technique, not only for morphologic visualizations, but also for the assessment of ventricular function, the thoracic vasculature and hemodynamics [6, 10, 11]. Since the care of patients with CHD has to be provided immediately after the birth to ensure appropriate and well-timed interventions, reliable and accurate CMR techniques for assessment of the thoracic vasculature in small pediatric patients are needed. However, imaging of neonates and young children might be challenging due to their body size, higher heart and respiratory rate, as well as limited cooperation levels, which would require general anesthesia. Therefore, further optimization of current CMRA approaches to achieve the best diagnostic quality is highly desirable. In this regard, higher field strengths might be favorable in small children, as due to the higher resonance frequencies an improved fat-saturation, and due to the longer T1 relaxation times a higher contrast-to-noise ratio can be achieved [12, 13]. To date, two main contrast-enhanced CMRA approaches are broadly used for assessment of the thoracic vasculature in CHD patients: standard time resolved multiphase first-pass CMRA and steady-state CMRA with high spatial resolution [14–16]. These two CMRA approaches have been compared regarding their diagnostic value for the accurate assessment of thoracic vasculature in adolescents and adult patients [15, 17, 18]. However, there are still no studies investigating the diagnostic utility of steady-state CMRA for the assessment of thoracic vasculature in sedated pediatric patients at 3 T. Also, the implementation of a robust fat suppression could increase the diagnostic utility of steady-state CMRA. In this respect, the implementation of the modified Dixon (mDixon) fat suppression method might be advantageous due to its chemical shift based uniform fat suppression and robustness to implants-induced artifacts [19–24].

Therefore, the aim of this study was to evaluate the diagnostic utility of a novel high-resolution electrocardiogram (ECG)- and navigator-gated, free-breathing steady-state CMRA using the mDixon method for fat suppression and compressed sensing in comparison to the standard, free breathing multiphase first-pass CMRA for the assessment of thoracic vasculature in pediatric CHD patients at 3 T.

## Methods

### Study cohort

This retrospective study was approved by the local institutional review board that waived written informed consent. From September to December 2020, consecutive pediatric patients with CHD, who underwent dedicated 3T CMR in deep sedation, were identified and included in this study. There were no exclusion criteria regarding the type of CHD or previous surgical procedures/interventions.

### Cardiovascular magnetic resonance

All CMR examinations were performed on a clinical whole-body 3 T CMR system (Ingenia Elition X, Philips Healthcare, Best, The Netherlands). For signal reception, a 32-channel torso coil (31 patients) or an 8-channel pediatric torso coil (one patient) with digital interface were used. The standard CMR protocol included ECG-gated steady state free-precession cine images in standard orientations (short-axis, two-chamber, four-chamber, left ventricular outflow tract, right ventricular outflow tract, transversal and coronal), phase contrast velocity measurements depending on the underlying CHD (most commonly: ascending aorta, main pulmonary artery, right and left pulmonary artery as well as superior and inferior vena cava), and late gadolinium enhancement (LGE).

#### First-pass CMRA

For first-pass CMRA, a standard 3-dimensional (3D) multiphase spoiled gradient echo CMRA sequence without respiratory motion compensation or ECG-triggering was used. In all patients, a native followed by three dynamic phases after injection of a gadolinium-based contrast agent at a dose 0.1 mmol per kg body weight and flow rate of 1.5 ml/sec were acquired (gadobutrol, Gadovist, Bayer Healthcare, Berlin, Germany).

#### Steady-state mDixon CMRA

For steady-state CMRA, a single phase mDixon sequence with respiratory navigator (end-expiration) and ECG-gating (end-diastolic) during slow infusion of the same contrast agent (0.1 mmol per kg body weight) using a flow rate of 0.1–0.3 ml/sec was performed. 19 k-space lines were acquired per cardiac cycle. The mDixon sequence was an unbalanced fast gradient echo sequence that achieves fat suppression through chemical shift-based water-fat separation [21–23]. Furthermore, compressed sensing (factor 6) was used to accelerate the acquisition of the mDixon steady-state CMRA. The employed compressed sensing technique was based on a combination of compressed sensing and parallel imaging using SENSE (Compressed SENSE, Philips Healthcare).

Acquisition of both CMRA sequences was three-dimensional and obtained in the coronal plane covering the chest, the neck and the upper abdomen. No breath holds were performed in acquisition of both, steady-state and first-pass CMRA. Detailed sequence parameters of both CMRA sequences are given in Table 1.

**Table 1:** Acquisition parameters of contrast-enhanced first-pass cardiovascular magnetic resonance angiography (CMRA) and modified Dixon (mDixon) steady-state CMRA

Parameter	First-pass CMRA	mDixon steady-state CMRA
Time of echo (msec)	1.60	1.93
Time of repetition (msec)	5.0	5.1
Total scan duration (min)	1:02 (15.2 s./per dynamic)	1:56
Acquired voxel size (mm)	1.7 × 1.7 × 3.4	1.20 × 1.20 × 2.40
Reconstructed voxel size (mm)	1.61 × 1.61 × 1.7	0.62 × 0.62 × 1.2
Turbo field echo factor	–	19
Field of view (mm)	360 × 360 × 139	240 × 240 × 96
Matrix (slices)	212 × 212 × 82	200 × 199 × 80
Compressed sensing	No	Yes, factor 6
Parallel imaging factor	Yes. P reduction (RL) 4, S reduction (AP) 1	No
Slice orientation	Coronal	Coronal
Number of slices	82	80
Fat suppression technique	Subtraction	mDixon
Flip angle	30	20
Cardiac synchronization device	−	ECG
Respiratory compensation	−	Navigator respiratory compensation gating. Gating window 2 (7)
Maximum intensity projection (slice thickness/gap, mm)	3.4/1.7	4/2
Acquisition mode	Cartesian	Cartesian

*ECG*, electrocardiogram; *AP*, Anterior Posterior; *RL*, Right-left

### Image analysis

Image analysis was performed by an experienced board-certified radiologist with 9 years of CMR experience (J.A.L., reader 1) and a radiologist with 3 year of CMR experience (N.M., reader 2), both blinded to the clinical information. For image analysis, a commercially available software (IMPAX EE R20, Agfa Healthcare, Mortsel, Belgium) was used. Image quality, blood-to-tissue contrast, the quality of fat suppression of the mDixon steady-state CMRA as well as quantitative measurements of thoracic vasculature were evaluated. For the qualitative and quantitative image analysis all dynamic phases of the first-pass CMRA and the water-only images of mDixon were used.

### Image quality assessment

Visual assessment of image quality for both CMRA types was performed by both readers in consensus for each pre-defined vessel separately. Image quality assessment was based on anatomical delineations, vessel sharpness, as well as breathing/motion and flow artifacts at the level of measurement by using a 5-point Likert scale, with 5 = excellent image quality (excellent delineation of vessel borders with no artifacts resulting in high diagnostic confidence); 4 = good image quality (good delineation of vessel borders with slight artifacts resulting in good diagnostic confidence); 3 = intermediate image quality (little blurring of the vessel borders, some artifacts resulting in decreased diagnostic confidence); 2 = poor image quality (severe blurring of the vessel borders and/or artifacts); 1 = non-diagnostic image quality (vessel borders are non-identifiable with severe artifacts). Pre-defined assessment points were as follows: ascending aorta, main pulmonary artery/conduit, all pulmonary arteries and veins, superior and inferior vena cava. For image quality analysis, readers had access to the maximum intensity projections as well as source data for both CMRA types. Additionally, the presence of fat-water separation artifacts of mDixon steady-state CMRA was analyzed.

### Blood-to-tissue contrast and quality of fat suppression

Blood-to-tissue contrast was assessed separately by both readers and measured as the ratio of signal intensity of the area of interest (blood pool of the tubular ascending aorta at mid-level/ at the level of pulmonary bifurcation) and the skeletal muscle (paraspinal muscles at the same level) in the water images of mDixon steady-state CMRA and the last phase of first-pass CMRA, as fundamental differences between first-pass CMRA and steady-state techniques regarding the contrast can be expected. Quality of fat suppression of mDixon steady-state CMRA was assessed in consensus by both readers using a score from 0 to 2, where 0 corresponds to no fat suppression, 1 corresponds to insufficient/inhomogeneous fat suppression, and 2 corresponds to sufficient/ homogeneous fat suppression.

### Quantitative measurements of thoracic vasculature

Measurements of vessel diameters were conducted separately by both readers at the same pre-defined positions for each CMRA type, independent of image quality. Measurements were performed on multiplanar reconstructed images based on the recommendations of the Society for Cardiovascular Magnetic Resonance (SCMR) for obtaining CMR values in adults and children by using the inner diameter approach [25, 26]. For each point of measurement, the average of two orthogonal measurements was used for final analysis. The pre-defined positions were as follows: ascending aorta and descending aorta at the level of pulmonary bifurcation, main pulmonary artery/conduit (distal of the pulmonary valve in the middle of the pulmonary trunk), right and left pulmonary artery (in case of present Glenn anastomosis distal of the anastomotic area), superior vena cava (in case of present Glenn anastomosis proximal the anastomotic area), right superior and inferior pulmonary vein, left superior and inferior pulmonary vein (1 cm distal of the atrial ostium). All measurements were performed in the appropriate dynamic phase of the first-pass CMRA and on the water images of the mDixon steady-state CMRA. Non-diagnostic images were excluded from analysis.

### Diagnostic utility

The assessment of diagnostic utility of each CMRA type was performed in consensus by both readers. Both CMRA types were separately analyzed in order to find any vascular abnormalities (e.g., coronary artery anomalies, aortopulmonary collateral arteries, stenotic areas), which could be identified and/or sufficiently assessed exclusively on either one of the CMRA types.

### Statistical analysis

Statistical analysis was performed using commercially available software SPSS ( version 25, Statistical Package for the Social Sciences, International Business Machines, Inc., Armonk, New York, USA or Prism 8 (GraphPad Software, San Diego, California, USA). Data are presented as mean ± standard deviation or as absolute frequency, as appropriate. Data were checked for normal distribution using Shapiro-Wilk test. Paired Student t test was used to compare the quantitative measurements on both CMRA sequences. The Wilcoxon matched pair test was used to evaluate the differences regarding the image quality. Overall image quality for each subject for each CMRA type was also calculated and expressed as a mean value ± standard deviation. Interobserver reproducibility was assessed using intraclass correlation coefficients (ICCs) and the Bland-Altman method. Furthermore, agreement between individual sets of measurements was performed with the Bland-Altman method. The level of statistical significance was set to P < 0.05.

## Results

### Cohort characteristics

A total of 32 pediatric patients with CHD were included in this study (13 females, 3.3 ± 1.7 years, body mass index 14.6 ± 1.5 kg/m^2^). All patients had clinical indications for CMR and underwent CMR while under deep sedation. There were no complications related to CMR examination or sedation. The baseline characteristics, primary cardiac diagnosis and indications for CMR are provided in Table 2.

**Table 2:** Baseline characteristics of included patients with congenital heart disease

Patient	Age (years)	Sex	Primary diagnosis	Indication for CMR
1	2	M	HRHS	Follow up before Fontan procedure
2	3	M	Shone-Syndrome	Follow up before Fontan procedure
3	2	M	DIRV, TGA	Follow up before Fontan procedure
4	5	M	ASD II, Wolf-Hirschhorn-Syndrome	Follow-up
5	4	F	ISTA, PVS	Follow-up
6	3	M	Tricuspid atresia Ib, restrictive VSD, PFO, PVS	Follow up before Fontan procedure
7	6	M	Pulmonary atresia	Follow up
8	5	F	ccTGA, VSD, ASD, pulmonary atresia	Follow up before Fontan procedure
9	5	F	TOF	Follow up
10	6	M	AVSD, BAV, PAPVD	Follow up before Fontan procedure
11	4	F	ccTGA, ISTA, VSD, ASD, AAH	Follow up
12	1	M	TGA, VSD	Evaluation before Switch operation
13	4	F	HLHS	Follow up before Fontan procedure
14	2	F	DORV, TGA, PVS	Follow up before Fontan procedure
15	3	F	HLHS	Follow up before Fontan procedure
16	3	M	HLHS, restrictive foramen ovale	Follow up before Fontan procedure
16	1	M	VSD (II)	Follow up
18	4	M	DILV	Follow up before Fontan procedure
19	3	M	HLHS, ASD	Follow up before Fontan procedure
20	4	F	DILV, L-TGA	Follow up before Fontan procedure
21	3	M	HLHS, ISTA	Follow up before Fontan procedure
22	2	F	HLHS	Follow up before Fontan procedure
23	3	F	HLHS	Follow up before Fontan procedure
24	3	M	HLHS	Follow up before Fontan procedure
25	1	M	Critical aortic stenosis, mitral valve insufficiency	Follow up after surgery/intervention
26	5	M	ccTGA, VSD, dextrocardia, RAoB, PVS	Follow up before surgery
27	8	M	Superior SVASD, PAPVD	Imaging, follow-up
28	2	F	DORV, HLVS, AVSD, PVS	Follow up before Fontan procedure
29	4	F	DORV, TGA, VSD, HLHS, ASD, PVS, LSVS, PDA	Follow up before surgery
30	3	M	HLHS	Follow up before Fontan procedure
31	1	M	Situs solitus abdominalis, heterotaxia with dextrocardia, AVSD, Azygos continuation	Follow up before surgery
32	0	F	Multiple VSDs	Follow up before surgery

CMR, cardiovascular magnetic resonance; HRHS, hypoplastic right heart syndrome; DIRV, double inlet right ventricle; TGA, transposition of the great arteries; ASD, atrial septal defect; ISTA, aortic isthmus stenosis; DORV, double outlet right ventricle; VSD, ventricular septal defect; PFO, patent foramen ovale; TOF, tetralogy of Fallot; AVSD, atrioventricular septal defect; BAV, bicuspid aortic valve; PAPVD, partial anomalous pulmonary venous drainage; AAH, aortic arch hypoplasia; HLHS, hypoplastic left heart syndrome; DILV, double inlet left ventricle; SVASD, Sinus venosus atrial septal defect; PVS, pulmonary valve stenosis

### Image quality assessment

**Fig. 1:**
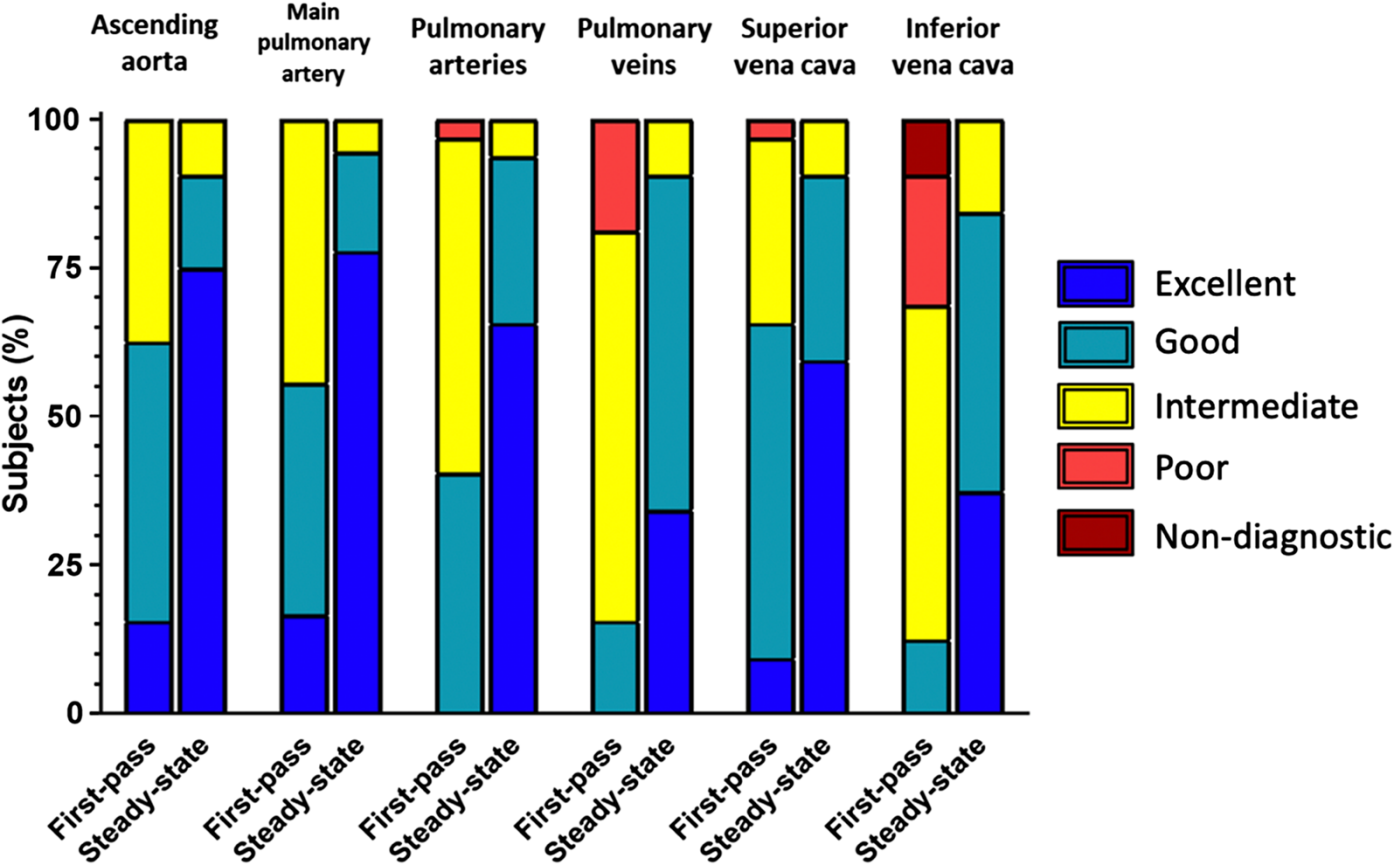
Bar plots of image quality scores of first-pass cardiovascular magnetic resonance angiography (CMRA) and mDixon steady-state CMRA using a 5-point Likert grading scale

**Fig. 2:**
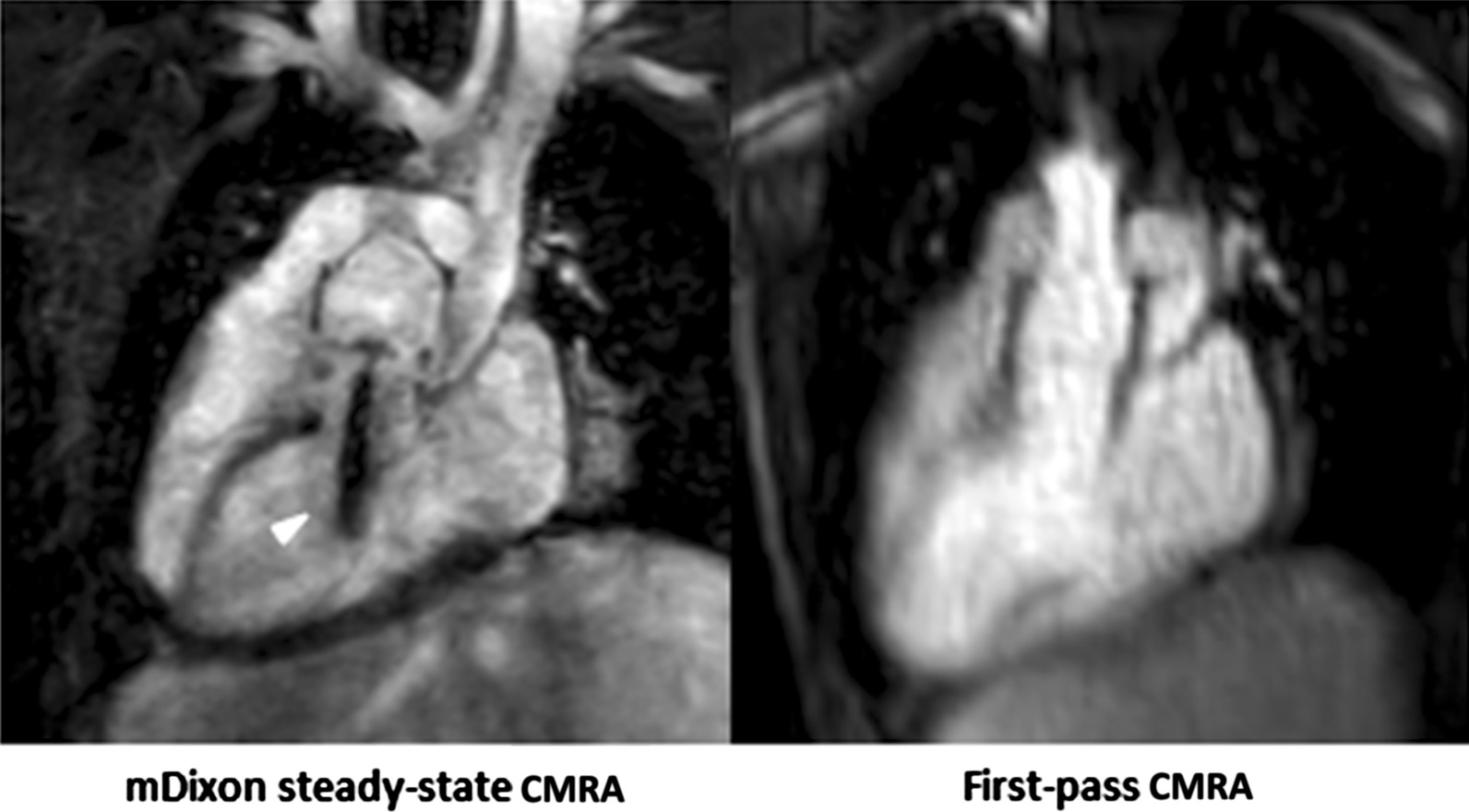
Representative images of a 5-year-old boy with congenitally corrected transposition of great arteries, ventricular septal defect, subpulmonic stenosis, dextrocardia after double switch procedure with Lecompte maneuver, resection of left ventricular outflow obstruction and patch closure of ventricular septal defect. mDixon steady-state CMRA (water-only reconstruction) showed fat-water separation artifact caused by stenosis of left ventricular outflow compared to first-pass CMRA (arrowhead)

Steady-state mDixon CMRA demonstrated significantly higher image quality compared to the first-pass CMRA regarding all investigated features. The most apparent difference of image quality was evident at the level of inferior vena cava (4.2 ± 0.7 vs. 2.7 ± 0.8; P < 0.001) followed by pulmonary arteries (4.6 ± 0.3 vs. 3.4 ± 0.6; P < 0.001) and veins (4.3 ± 0.6 vs. 2.9 ± 0.6; P < 0.001). The overall image quality of steady-state mDixon CMRA was also significantly higher compared to first-pass CMRA (4.5 ± 0.5 vs. 3.3 ± 0.5, P < 0.001). The highest image quality score for both types of CMRA was achieved at the level of ascending aorta with mean score of 4.7 ± 0.7 and 3.8 ± 0.7 for steady-state mDixon and first-pass CMRA, respectively. The detailed results of image quality assessment are given in the Table 3, see also Fig. 1. In 3/32 (9%) cases, fat-water separation artifacts caused by stenosis were observed in mDixon steady-state CMRA (see also Fig. 2).

**Table 3:** Image quality assessment of the thoracic vasculature in pediatric patients with congenital heart disease (CHD) according to a 5-point Likert grading scale

Thoracic vessels	Image quality assessment		P value
	First-pass CMRA	mDixon steady-state CMRA	
Ascending aorta	3.8 ± 0.7	4.7 ± 0.7	< 0.001
Main pulmonary artery /conduit	3.7 ± 0.8	4.7 ± 0.6	< 0.001
Pulmonary arteries	3.4 ± 0.6	4.6 ± 0.3	< 0.001
Pulmonary veins	2.9 ± 0.6	4.3 ± 0.6	< 0.001
Superior vena cava/Glenn anastomosis	3.7 ± 0.8	4.5 ± 0.7	< 0.001
Inferior vena cava	2.7 ± 0.8	4.2 ± 0.7	< 0.001
Overall image quality	3.3 ± 0.5	4.5 ± 0.5	< 0.001

All data are mean ± standard deviation

### Blood-to-tissue contrast and quality of fat suppression

Even if not significant, steady-state mDixon CMRA demonstrated higher blood-to-tissue contrast compared to the venous phase of first-pass CMRA by both readers with mean values of 7.85 ± 4.75 vs. 6.35 ± 2.23 for reader 1 (P = 0.133) and 7.69 ± 4.50 vs. 6.20 ± 2.47 for reader 2 (P = 0.138). Regarding fat suppression, there were only a few cases (2/32, 6%) with a slight insufficient fat suppression at the level of the neck.

### Quantitative measurements of the thoracic vasculature

**Fig. 3:**
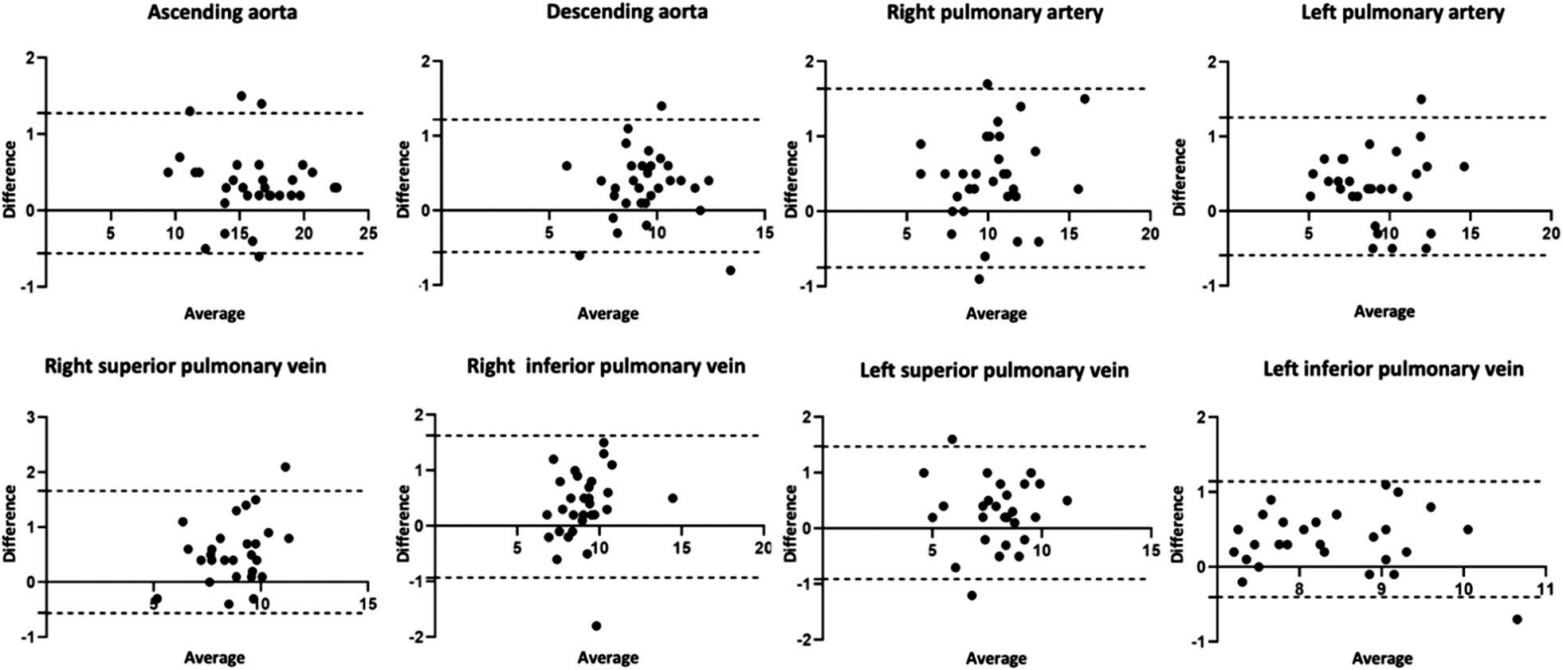
Bland-Altman plots show comparison of quantitative assessment of thoracic vessel diameters at different measurement points between first-pass and mDixon steady-state CMRA performed by reader 1.The mean value of measurements for both CMRA approaches is plotted on the x-axis and the difference between techniques is plotted on the y-axis. The solid black horizontal line plots the mean difference and the dotted black lines indicate 95% confidence interval for each point of measurement

First-pass CMRA showed significantly greater diameters at all measurement points for both readers with the only exception at the level of left superior pulmonary vein for reader 2 (P = 0.089). The overestimation of measurements of first-pass CMRA, was confirmed in Bland-Altman analysis for quantitative vascular measurements. For instance, for the reader 1 some representative measurements were as follows: ascending aorta: bias 0.36 ± 0.47 mm (95% limits of agreement (LOA) −0.56–1.28 mm); right pulmonary artery: bias 0.45 ± 0.61 mm (95% LOA − 0.75–1.64 mm); right superior pulmonary vein: bias 0.55 ± 0.57 mm (95% LOA − 0.56–1.67 mm); right inferior pulmonary vein: bias 0.35 ± 0.65 mm (95% LOA − 0.93–1.63 mm), see also Fig. 3. Detailed parameters of the thoracic vasculature measurements at all measurement points are given in Table 4.

**Table 4:** Quantitative measurements of the thoracic vasculature of both readers on first-pass CMRA and mDixon steady-state CMRA at pre-defined measurement points

Measurement points	Reader 1			Reader 2		
	First-pass CMRA	mDixon Steady-state CMRA	P value	First-pass CMRA	mDixon Steady-state CMRA	P value
Ascending aorta (mm)	16.2 ± 3.3	15.8 ± 3.4	< 0.001	16.1 ± 3.3	15.8 ± 3.4	0.001
Descending aorta (mm)	9.6 ± 1.6	9.3 ± 1.7	< 0.001	9.6 ± 1.6	9.3 ± 1.6	< 0.001
Main pulmonary artery/conduit (mm)	17.1 ± 4.7	16.5 ± 4.3	0.012	17.1 ± 4.7	16.7 ± 4.4	0.007
Right pulmonary artery (mm)	10.4 ± 2.4	9.9 ± 2.3	< 0.001	10.5 ± 2.5	9.9 ± 2.4	< 0.001
Left pulmonary artery (mm)	9.3 ± 2.4	8.9 ± 2.4	< 0.001	9.4 ± 2.5	8.9 ± 2.5	< 0.001
Superior vena cava/Glenn anastomosis (mm)	12.1 ± 2.6	11.7 ± 2.7	0.007	12.0 ± 2.6	11.7 ± 2.7	0.004
Right superior pulmonary vein (mm)	8.9 ± 1.6	8.4 ± 1.4	< 0.001	8.9 ± 1.5	8.4 ± 1.4	< 0.001
Right inferior pulmonary vein (mm)	9.2 ± 1.5	8.9 ± 1.5	0.006	9.3 ± 1.7	8.9 ± 1.5	0.001
Left superior pulmonary vein (mm)	8.0 ± 1.6	7.7 ± 1.6	0.023	8.1 ± 1.6	7.9 ± 1.6	0.089
Left inferior pulmonary vein (mm)	8.6 ± 0.9	8.2 ± 0.9	< 0.001	8.6 ± 1.0	8.3 ± 1.0	0.023

All data are mean ± standard deviation

**Fig. 4:**
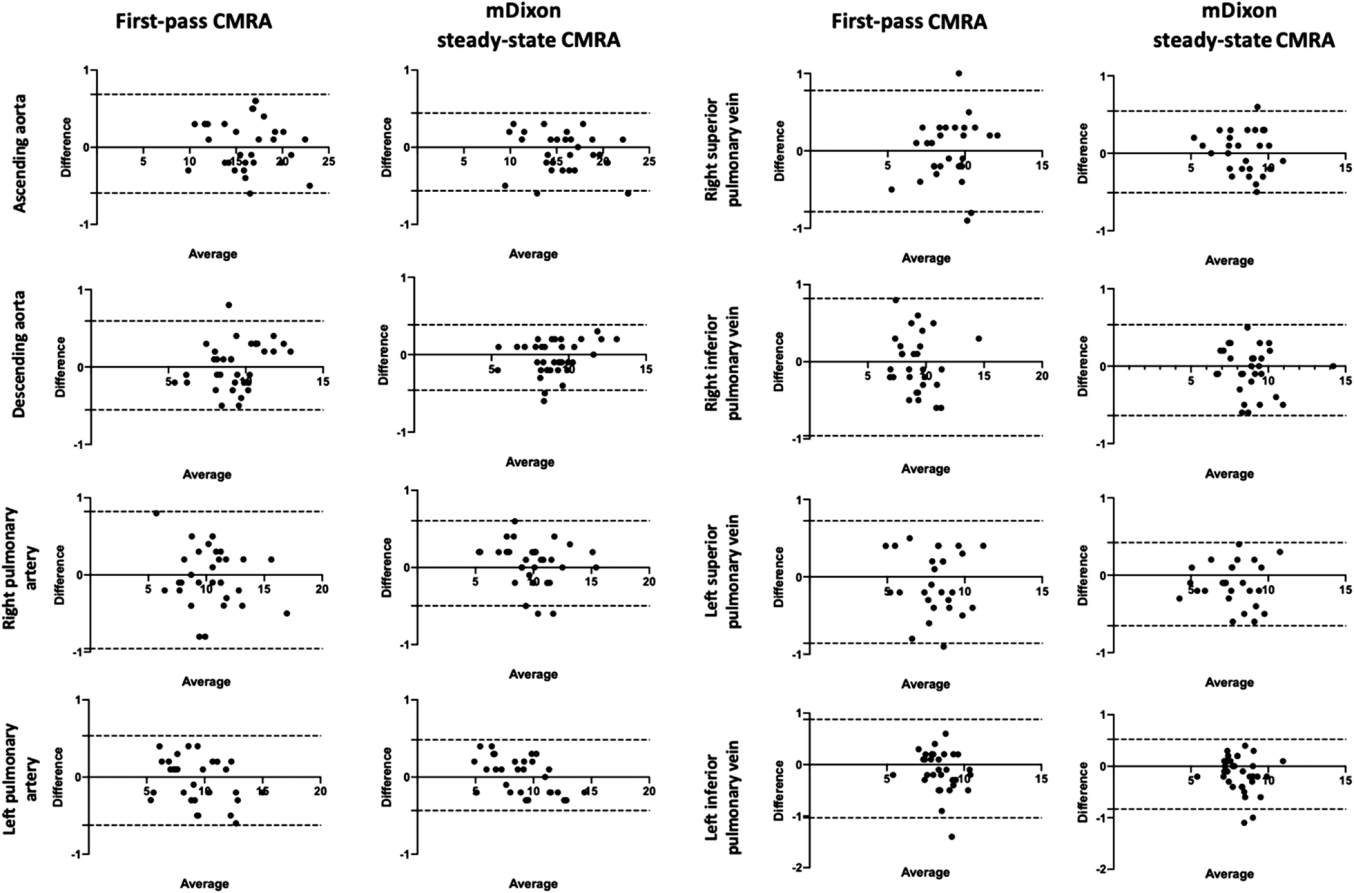
Bland-Altman plots show interobserver reproducibility for mDixon steady-state CMRA compared to first-pass CMRA for ascending and descending aorta, right and left pulmonary arteries as well as right and left pulmonary veins. The mean value of measurements for both readers is plotted on the x-axis and the difference between two readers is plotted on the y-axis. The solid black horizontal line plots the mean difference and the dotted black lines indicate 95% confidence interval for each point of measurement

Regarding interobserver reproducibility, Bland-Altman analysis showed a lower bias and closer LOA for mDixon steady-state CMRA compared to first-pass CMRA, with the most apparent differences at the level of pulmonary arteries and veins, e.g., right pulmonary artery: mDixon steady-state CMRA bias − 0.05 ± 0.28 mm (95% LOA: − 0.49–0.61 mm), first-pass CMRA bias − 0.07 ± 0.45 mm (95% LOA: − 0.96–0.82 mm); right superior pulmonary vein: mDixon steady-state CMRA bias 0.02 ± 0.27 mm (95% LOA: − 0.51–0.55 mm), first-pass CMRA bias − 0.03 ± 0.39 mm (95% LOA: -0.79–0.78 mm); right inferior pulmonary vein: mDixon steady-state CMRA bias − 0.05 ± 0.29 mm (95% LOA: − 0.64–0.53 mm), first-pass CMRA bias − 0.07 ± 0.45 mm (95% LOA: − 0.96–0.82 mm), see also Fig. 4. Both CMRA types revealed high ICCs at all measurement points with slightly higher ICCs for mDixon steady-state CMRA (> 0.92) compared to first-pass CMRA (> 0.86) and the highest difference at the level of pulmonary veins (see also Table 5**)**.

**Table 5:** Intraclass correlation coefficients (ICCs) of both CMRA methods at all measurement points

Measurement points	First-pass CMRA	P value	mDixon steady-state CMRA	P value
Ascending aorta	0.995	< 0.001	0.998	< 0.001
Descending aorta	0.984	< 0.001	0.987	< 0.001
Main pulmonary artery/Conduit	0.993	< 0.001	0.995	< 0.001
Right pulmonary artery	0.983	< 0.001	0.986	< 0.001
Left pulmonary artery	0.993	< 0.001	0.993	< 0.001
Superior vena cava /Glenn anastomosis	0.987	< 0.001	0.995	< 0.001
Right superior pulmonary vein	0.961	< 0.001	0.972	< 0.001
Right inferior pulmonary vein	0.961	< 0.001	0.976	< 0.001
Left superior pulmonary vein	0.968	< 0.001	0.972	< 0.001
Left inferior pulmonary vein	0.862	< 0.001	0.924	< 0.001

### Diagnostic utility

**Fig. 5:**
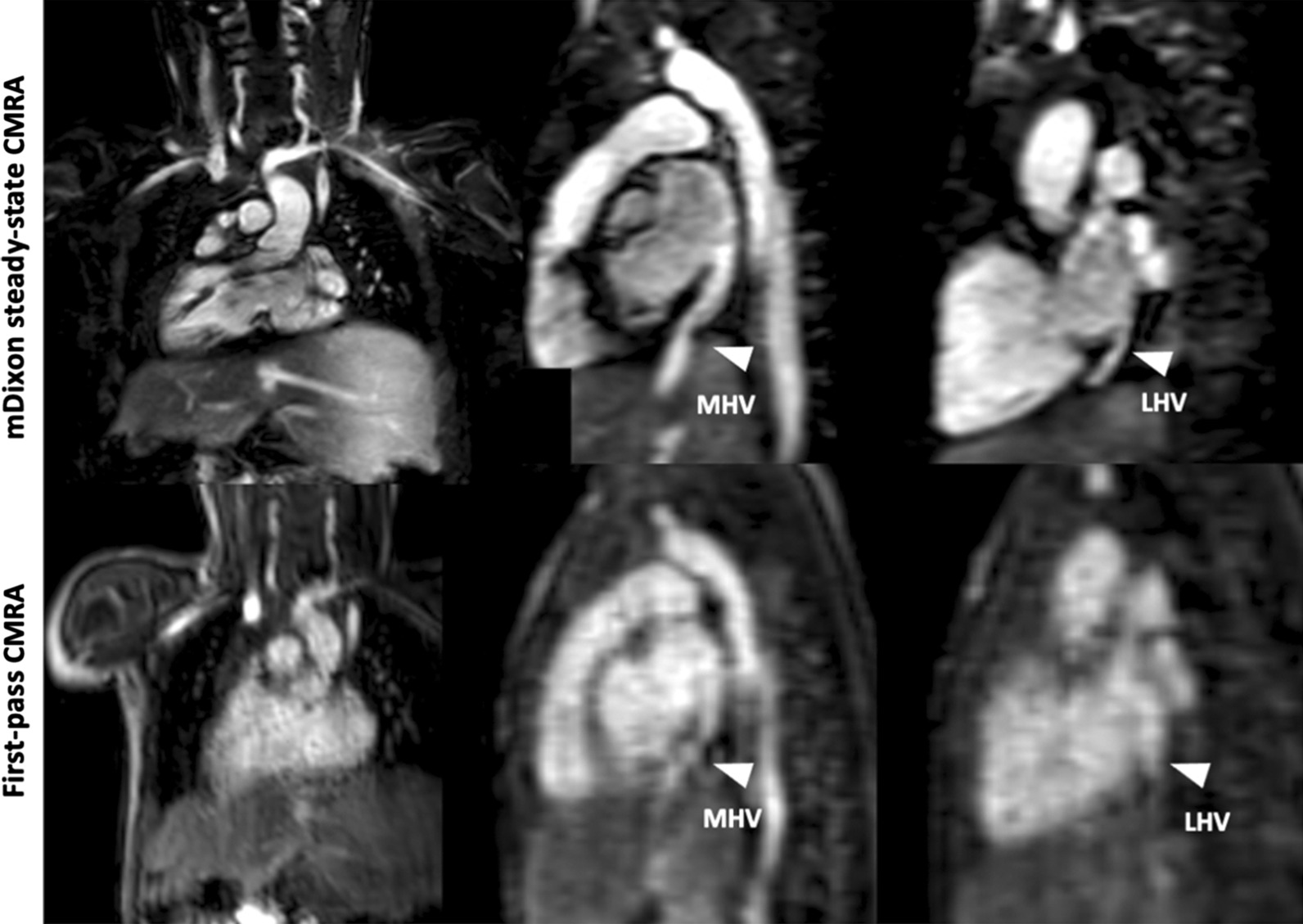
Representative images of a 1-year-old boy with heterotaxy, dextrocardia and anomalous hepatic venous drainage. Right (RHV) and middle hepatic (MHV) veins drain into right-sided atrium and left hepatic vein (LHV) drains into left-sided atrium. mDixon steady-state CMRA (water-only reconstruction) demonstrates good image quality with perfect fat suppression, allowing for better delineation of vessels and their origins compared to first-pass CMRA

**Fig. 6:**
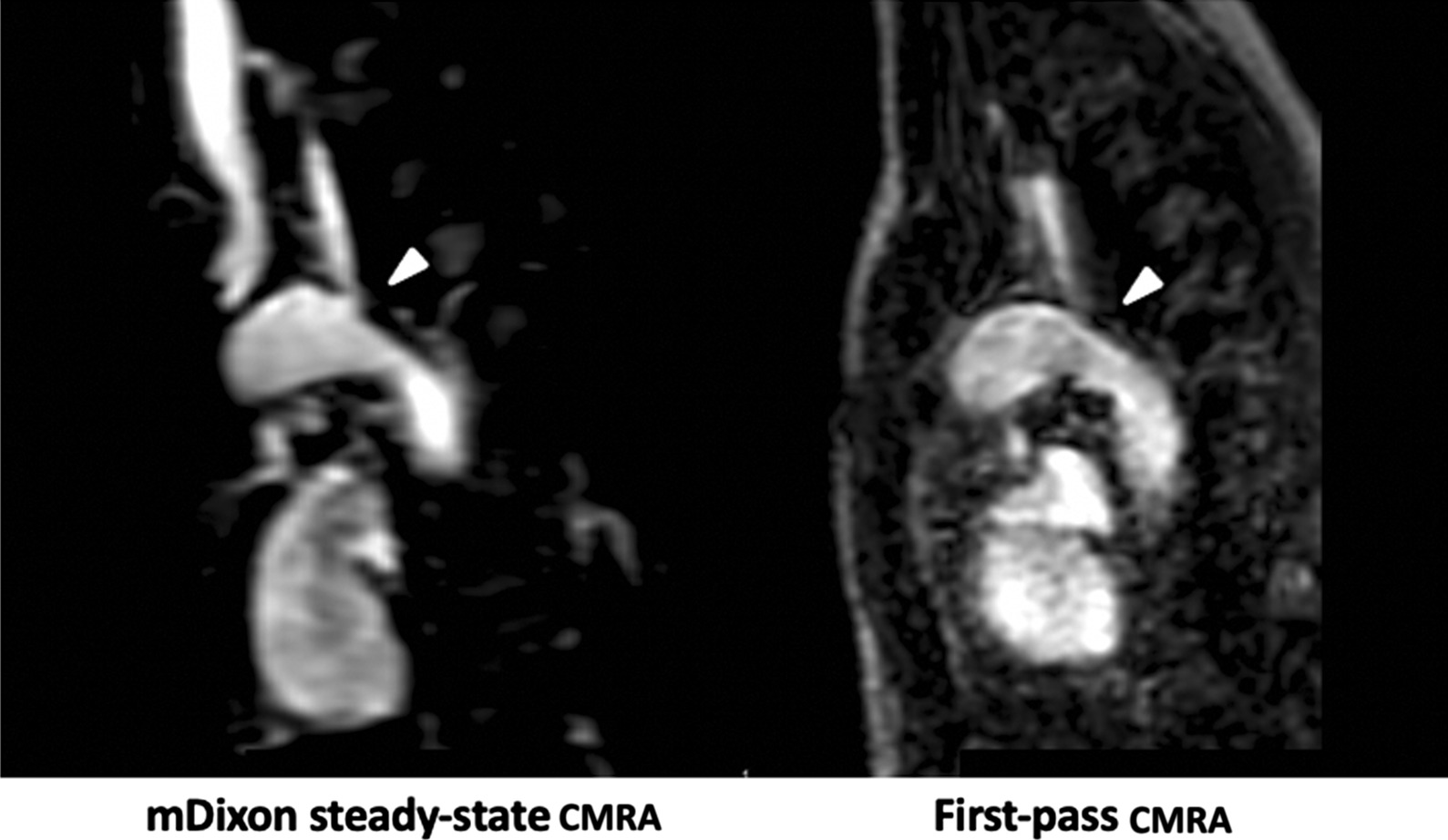
Representative images of a 3-year-old boy with congenital heart disease and subclavian artery stenosis (arrowheads). mDixon steady-state CMRA (water-only reconstruction) demonstrates higher image quality and spatial resolution allowing for better delineation of vessels and their origins for precise assessment of stenotic areas compared to first-pass CMRA

**Fig. 7:**
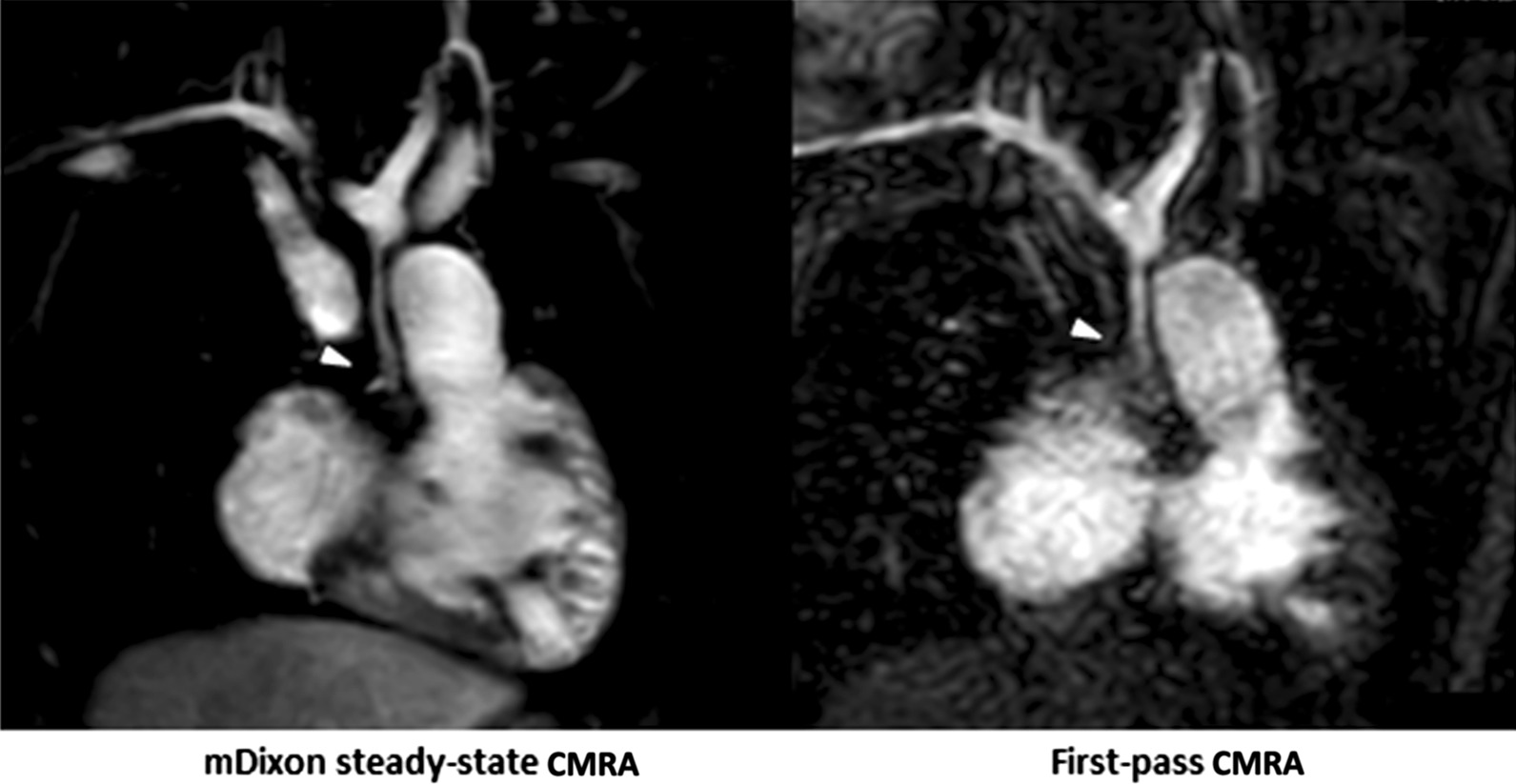
Representative images of a 3-year-old boy with hypoplastic left heart syndrome with retrograde coronary arterial perfusion from hypoplastic ascending aorta after multiple cardiac surgeries and interventions. mDixon steady-state CMRA (water-only reconstruction) demonstrates higher image quality and spatial resolution with perfect fat suppression, allowing for precise delineation and assessment of proximal coronary arteries and their origins compared to first-pass CMRA (arrowheads)

**Fig. 8:**
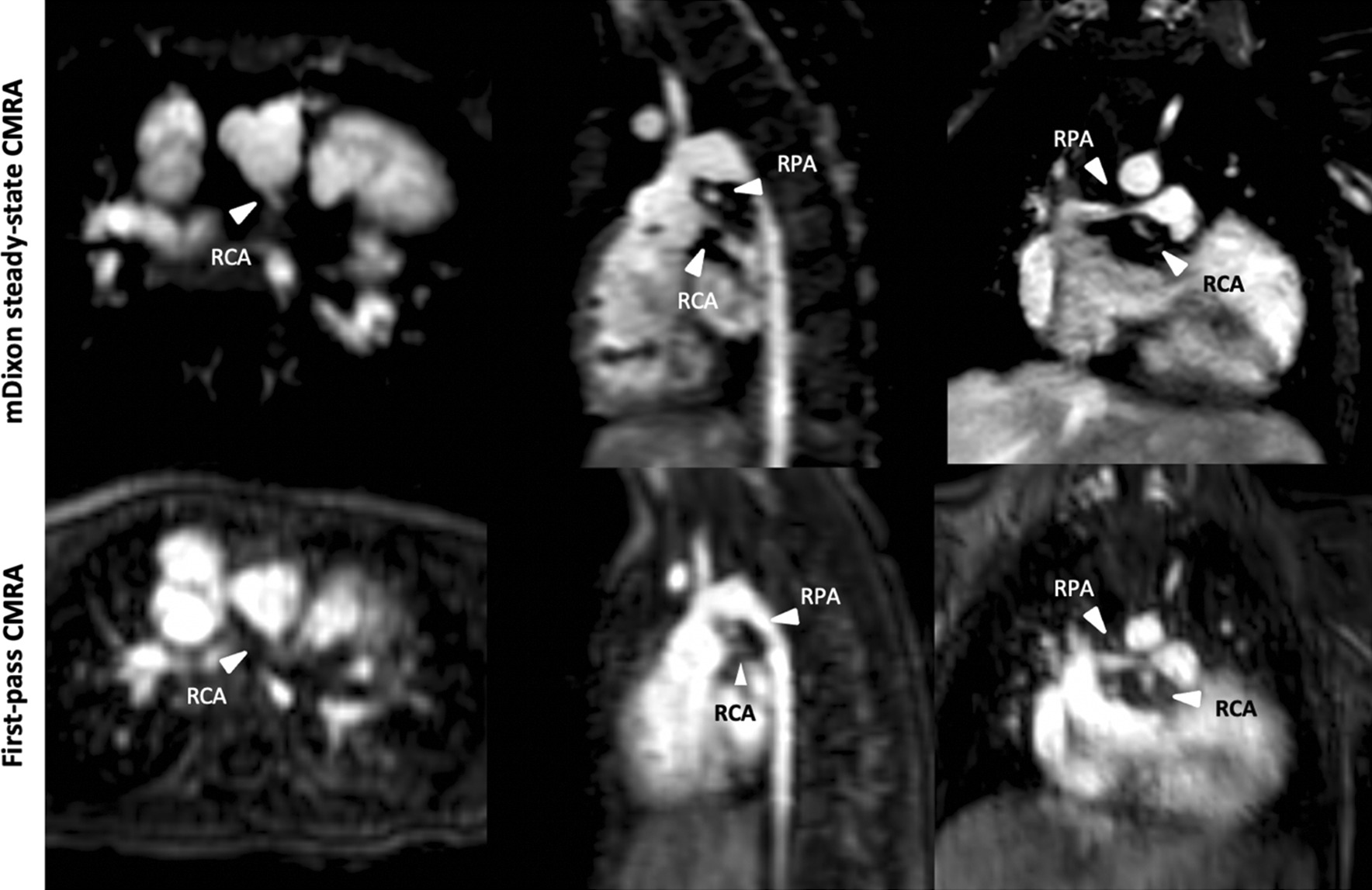
Representative images of a 4-year-old girl with congenitally corrected transposition of the great arteries, hypoplastic aortic arch, ventricular and atrial septal defects and aortic isthmus stenosis. Image acquisition was performed after double switch procedure, aortic arch reconstruction, resection of aortic isthmus stenosis and coronary arteries reinsertion (1LCx 2R). Images demonstrate stenosis of the right pulmonary artery and extremely close anatomical proximity of the right coronary ostium to the right pulmonary artery due to high spatial resolution and perfect fat suppression

**Fig. 9:**
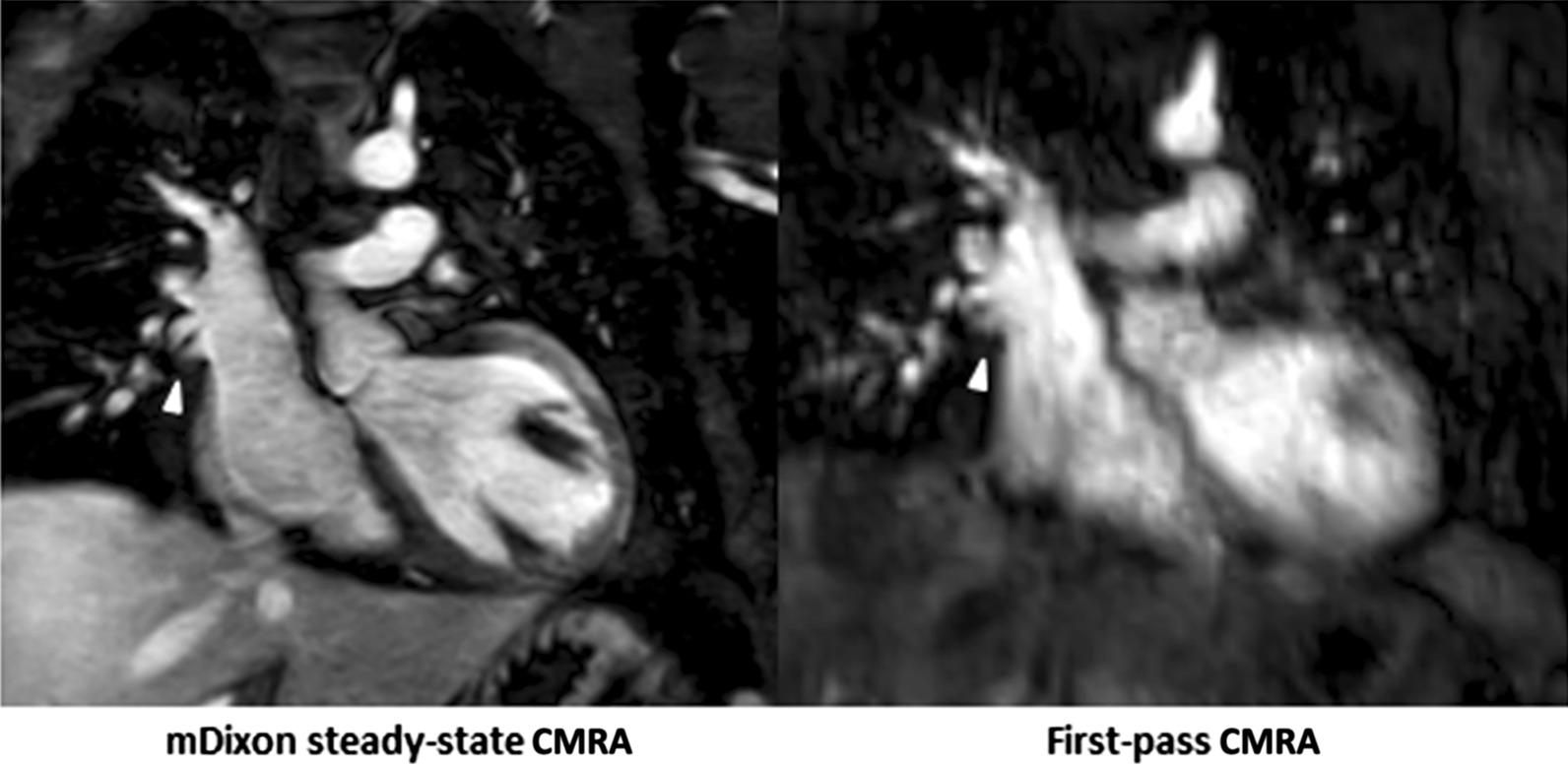
Representative images of an 8-year-old boy with partial anomalous venous return. mDixon steady-state CMRA (water-only reconstruction) demonstrates high image quality without blurring, enabling better vessel delineations compared to first-pass CMRA (see arrowhead)

**Fig. 10:**
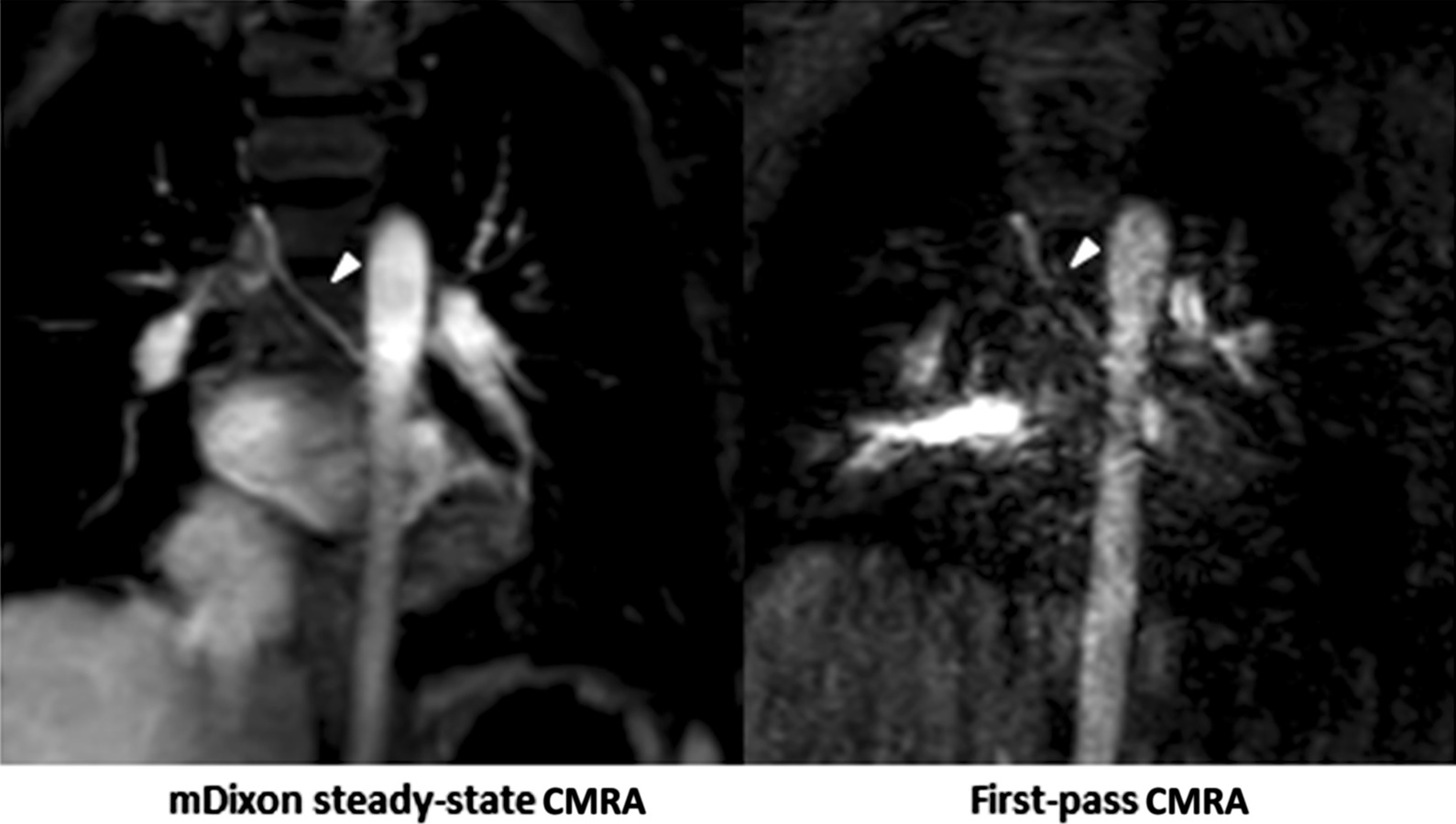
Representative images of a 2-year-old boy with hypoplastic right heart syndrome, tricuspid valve hypoplasia and pulmonary atresia after Glenn procedure. mDixon steady state CMRA (water-only reconstruction) allows for a better delineation of aortopulmonary collateral artery (arrowhead) due to higher spatial resolution and less artifacts without blurring and perfect fat suppression compared to first-pass CMRA

In 9/32 (28%) patients, mDixon steady-state CMRA provided 10 additional findings, which could not be identified and/or sufficiently assessed in the first-pass CMRA. These findings included: anomalous hepatic venous drainage (see Fig. 5), subclavian artery stenosis (see Fig. 6), abnormalities of the coronary arteries (see Figs. 7 and 8), partial anomalous venous return (see Fig. 9), and aortopulmonary collateral arteries (see Fig. 10).

## Discussion

In this study we compared a novel free breathing respiratory navigator and ECG-gated mDixon steady-state CMRA with compressed sensing to a standard, also free breathing, multiphase first-pass CMRA for the assessment of the thoracic vasculature in sedated pediatric CHD patients at 3 T. The main findings of our study are: (1) mDixon steady-state CMRA revealed a significantly better image quality than first-pass CMRA at all measurement points; (2) the quantitative vessel diameters were overestimated in first-pass CMRA compared to mDixon steady-state, which also showed a higher interobserver agreement; (3) mDixon steady-state CMRA revealed a high blood-to-tissue contrast, which was comparable to the standard first-pass CMRA, and a perfect fat suppression in almost all cases; (4) mDixon steady-state CMRA demonstrated high diagnostic utility by providing valuable additional information about vascular abnormalities, which could not be identified and/or sufficiently assessed in first-pass CMRA.

Current technical improvements in CMR techniques, as well as a broader implementation of scanners with higher magnetic field strengths, allow for an optimization of the present standard CMRA techniques [27]. Basically, two main contrast enhanced CMRA approaches are currently implemented: first-pass CMRA and steady-state CMRA [27, 28]. First-pass CMRA enables the functional assessment of hemodynamics due to high temporal resolution. In sedated pediatric patients, neither respiratory compensation nor ECG gating are normally possible, which might lead to an impaired image quality. Furthermore, first-pass CMRA has a limited spatial resolution, leading to limited diagnostic value and confidence as well as misinterpretation in infants and little children, which is of great clinical importance. To overcome these limitations, steady-state CMRA has been broadly used in clinical practice as an additional CMRA technique. In contrast to first-pass CMRA, steady-state CMRA has a higher spatial resolution which enables a more accurate morphological assessment of the thoracic vasculature. The higher image quality of steady-state CMRA compared to the first-pass CMRA is well-known and sufficiently described in adults and adolescents with CHD [14, 15, 17, 18]. Nevertheless, CMR in infants and little children remains challenging, especially considering the increasing use of higher magnetic fields in the pediatric population.

The main finding of our study is that the image quality of mDixon steady-state CMRA is higher than that of first-pass CMRA. Difference in diagnostic quality was most evident at the level of the smaller thoracic vessels: pulmonary arteries and veins. On the one hand, this can be explained by the nature of steady state approach [14, 15], on the other hand by the applied mDixon method. The post-contrast mDixon technique, using chemical shift-based water-fat separation, has a high blood-to-tissue contrast due to a high signal intensity of the blood, independence of flow artifacts and the suppression of surrounding fat. First-pass techniques are usually acquired during maximum arterial intravascular enhancement and typically provide a very high blood-to-tissue contrast. In this regard, the at least a comparable blood-to-tissue contrast of the steady-state mDixon CMRA, which is furthermore not restricted to a short scan time, can be regarded as advantageous. More importantly, the mDixon techniques has a known insensitivity for B0 and B1 inhomogeneities, which makes it less sensitive to magnetic field inhomogeneities [20, 22, 29–31]. For instance, only in 3/32 (9%) cases, fat-water separation artifacts were present in mDixon steady-state CMRA, however, they had no influence on image interpretation [32]. Another strong advantage of the mDixon technique is a robust fat suppression even in difficult anatomical areas with a large field of view, also in the pediatric population [20, 24]. In fact, there were only a few cases with a slightly insufficient fat suppression at the neck level, which, however, had no influence on general image quality and diagnostic utility. Furthermore, a sufficient fat suppression is important in order to differentiate small vessels from surrounding fatty tissue. The presented sequence also was combined with compressed sensing, which allows for an even shorter acquisition time without decreasing the spatial resolution [33, 34].

Another finding of our study is that first-pass CMRA overestimates vessel diameters compared to steady-state CMRA. This finding is supported by previous data, which also demonstrated significant differences in vascular measurements in favor of first-pass CMRA [35]. This could be explained by pulsation and breathing artifacts resulting in blurring and poor vessel delineation, which is greater at the level of smaller vessels, such as pulmonary arteries and veins. Furthermore, it is known that patients with CHD have an abnormal cardiac anatomy and a different circulation physiology (e.g. pulsatile circulation in tetralogy of Fallot) [25]. Also, the absence of motion compensation as well as ECG-triggering additionally contributes to a decreased sharpness of cardiac and paracardiac structures. Above named factors lead to an impaired image quality and consequently to significant differences in vessel measurements (with an overestimation of vessel size in the first-pass CMRA). As a precise assessment of the thoracic vessel diameters is of clinical importance (e.g., for catheter intervention planning), at least the combination of these two approaches should be considered to avoid misinterpretations.

Finally, mDixon steady-state CMRA demonstrated high diagnostic utility by providing valuable additional information about vascular abnormalities above first-pass CMRA due to significantly higher image quality and spatial resolution as well as perfect fat suppression, enabling better delineation of small vascular structures [17]. For instance, mDixon steady-state CMRA allowed for more accurate delineation of the proximal coronary arteries in two patients (see also Figs. 7 and 8). In general, the robust fat suppression of mDixon CMRA enables better delineation of coronary arteries. For instance, this was crucial for procedural planning (stent implantation) in one patient with right pulmonary artery stenosis, considering the extremely close anatomical proximity of the right pulmonary artery and coronary artery (Fig. 8). Also, detection and imaging of additional aortopulmonary collateral arteries and partial anomalous pulmonary venous return are of clinical importance, as they have relevant influence on hemodynamics in CHD patients and, therefore, on procedural planning (Figs. 9 and 10). Furthermore, due to high contrast and spatial resolution, better delineation of vessels is possible, e.g., in 2 cases with subclavian artery stenosis, which was difficult to detect on first-pass CMRA due to small body size, motion and breathing artifacts (Fig. 6).

## Limitations

Our study has several limitations. The main limitation of the current study is a relatively small sample size. However, we could include a broad range of CHD and, therefore, different alterations caused by multiple interventions/surgery prior to CMR examination. This fact adds additional value to our study, enabling the general applicability of our study results. Furthermore, both CMRA sequences were compared to each other without a comparison to a reference standard (e.g., catheter angiography). However, this study was aimed to describe a new technical method for a high-resolution steady-state CMRA and was not designed to correlate different CMRA techniques with a reference standard for vessel visualization. The intention of this study was also not to advocate the use of only one CMRA technique alone, but to show the benefit of a combined CMRA approach. Another limitation of our study was that both readers were not blinded to the type of CMRAs, which is impossible, as an experienced reader will be able to differentiate the two included sequences based on their visual impression. Finally, the fact that CMRA techniques may vary across institutions can additionally limit the general applicability of our study results.

## Conclusions

Our study results support the clinical application of a novel high-resolution ECG- and navigator-gated mDixon steady-state CMRA with compressed sensing at 3 T in sedated pediatric CHD patients. The described sequence has a robust and reliable image quality with a high spatial resolution and an excellent fat suppression, insensitive to the magnetic field inhomogeneities, enabling simultaneous imaging of arterial and venous structures in a large field of view.

## Data Availability

The datasets used and/or analyzed during the current study are available from the corresponding author on reasonable request.

## Abbreviations

CHD: Congenital heart disease
CMR: Cardiovascular magnetic resonance
CMRA: Cardiovascular magnetic resonance angiography
ECG: Electrocardiogram
LGE: Late gadolinium enhancement
LOA: Limits of agreement
mDixon: Modified Dixon
